# Editorial

**Published:** 1847-03

**Authors:** 


### THE MEDICAL EXAMINER.

PHILADELPHIA, MARCH, 1847.

### LA LANCETTE CANADIENNE.

We have received the first and third numbers of a new Medical journal with this title, in the French language, published at Montreal (Canada) on the first and fifteenth of every month. The journal is edited and published by Dr. J. L. Leprohon, and from the ability displayed in the numbers before us, promises to be an able coadjutor in the good work of diffusing Medical knowledge. A considerable portion of the practitioners of Canada speak and write the French as their vernacular language, and to them such a publication must be particularly acceptable; and as many of the younger and more aspiring members of our profession in the United States pay more or less attention to it, they will find the perusal of such a journal to subserve the double purpose of furnishing them with much valuable information in the line of their profession, and of improving them in the knowledge of the language in which it is conveyed. We wish the enterprising editor a full realization of his warmest anticipations, whilst we promise ourselves the pleasure of occasionally gleaning from his labours for the benefit of our readers.

### NATIONAL MEDICAL CONVENTION.

From all that we can discover, the Medical Convention to be held in this City in May next is likely to be the largest body of the kind ever assembled. Delegates have already been appointed by most of the Colleges and chartered Medical Societies throughout the Union, and as the number of representatives from each body is determined by itself, without any suggestion or restriction emanating from any other quarter, many of them have chosen large delegations, especially the popular bodies. These latter, it seems to us, if the delegates vote per capita, will not only swallow up the schools, but, to use a bull, swallow themselves too—that is, the large number appointed by a few of them in the principal cities will out-vote all the rest. However, if wise counsels prevail so that they vote aright, and benefit accrues to the profession and the people, there will be none to complain.

The number appointed in this city, we believe, is as follows, viz:

| | | |
|---|---|---|
| By the | Philadelphia Medical Society, | twelve. |
| " | College of Physicians, - - | twelve. |
| " | University of Pennsylvania, - | three. |
| " | Jefferson Medical College, - | three. |
| " | Pennsylvania College, - - | three. |
| " | Franklin College, - - | three. |
| " | Northern Medical Association, - | five. |
| " | Philadelphia College of Medicine, | two—in all forty-three. |

---

CATALOGUES OF MEDICAL SCHOOLS.

Last year we collected from the Catalogues and Medical Journals published in the different sections of the United States, the number of students and graduates of the season, and published the whole in tabular form, and we are desirous of doing the same the present year. To enable us to perform this task accurately, we respectfully request of the deans or secretaries of the different institutions to forward to us their respective Catalogues as soon as published. Thus far we have received but four, viz: those of the Jefferson Medical College of Philadelphia, the University of Pennsylvania, of the University of Louisville (formerly Medical Institute,) and the Memphis Medical College of Tennessee.

From these it appears that the class of the Jefferson Medical College during the past Session numbered - - - - 493

| | |
|---|---|
| University of Pennsylvania - - - - - - - | 412 |
| University of Louisville - - - - - - - - | 348 |
| Memphis Medical College - - - - - - - | 55 |

---

NORTHERN MEDICAL ASSOCIATION OF PHILADELPHIA.

In a former number we mentioned that the physicians residing in the northern part of Philadelphia, including Germantown and Frankford, were about to form themselves into a society for the promotion of science and good-fellowship; since then, we understand that an association has been regularly organized under the title of "*The Northern Medical Association of Philadelphia*," and that on the 14th of January last the following gentlemen were duly elected officers for the present year:

*President*—Dr. Benjamin S. Janney.

*Vice-President*—Dr. Arnold Naudain.

*Counsellors*—Drs. Uhler, Yardley and Hatfield.

*Treasurer*—Dr. M. B. Smith.

*Secretary*—Dr. Remington.

*Reporting Secretaries*—Drs. Townsend and Bryan.

The association has been judicious in the selection of gentlemen for its officers who are greatly respected in this community, and under such favourable auspices the best results must follow its organization.

